# Physiological Observations on the Teeth

**Published:** 1858-08

**Authors:** F. E. St. John


### THE DENTAL REPORTER.

Vol. 1. AUGUST, 1858. No. 2.

### PHYSIOLOGICAL OBSERVATIONS ON THE TEETH.

BY PROF. F. E. ST. JOHN.

Aside from the disgusting odor and foul breath; the tormenting pain accompanied by its distracted days and sleepless nights, the dread disease that

"Ay mocks our groans!"

and fills the cup of human misery to its brim, an imperfect set of teeth are still a thing of dread. The noisome gasses arising from the decaying tooth, are carried at each inspiration to the lungs, contaminating every thing they touch, and sowing the seeds of disease, which, operating upon the great functions of life, consign the victim slowly but surely to an early and untimely grave. It is a lamentable fact that there are very few among the millions of this beautiful country who seem to understand that the teeth are among the most important of the organs of the human body, and they subject them to the roughest and most inhuman treatment; grinding with them, blocks of wood, bone, granite and hickory nuts, and subjecting them to the action of hot wator and cold, acids and alkalies, the disgusting juice of the more filthy tobacco, and the burning lava direct from the seething pot; of the lower "hades" in shape of "brandysmashes," or "gin cock tails," until the flinty structure gives way, and the indulger finally falls into the destroying angel's death-like grasp, a victim to his own ungoverned passions.

But this is not its worst feature; the parent, after thus wantonly destroying so important an organ, and spreading the results of its decay over every portion of his body until his very being is contaminated, and a broken constitution and often a half destroyed intellect tells too plainly how sadly he has disobeyed the laws of life, transmits this imperfect organization and diseased diathesis to his child, until is seen a verification of Holy Writ, in which " the sins of the parents are visited upon the third and fourth generations ;" and children, while in the possesion of their temporary teeth, are tortured with the toothache, occasioned by the carious organ.

It is truly deplorable that we should be thus early afflicted with decay of those organs which are formed of substances that enter into the formation of some of the hardest materials, as marble, bone-earth and fluor-spar ; and we see, even in youth, many instances in which the natural has to be replaced by the unnatural and more enduring enamel of the artist.

What is the cause of this ? It was not so in olden times, and it is not so in many other countries, in which the inhabitants live more in accordance with the laws of health. The primary cause is with the parents, they by excesses and total disregard of all the laws of life, have entailed upon the child a constitutional weakness, caused by a deficiency of the inorganic or earthy constituents which should form a portion of the child during its early existence. In order to un lerstand more fully what these deficiencies are, it will be necessary to examine briefly the structure and composition of the teeth. In structure, they are nearly allied to bone, both being supplied with certain earthy constituents and animal matter, which, by consolidation give form and strength to the organ.

The teeth are composed of three different substances, *cementum* or *crusia peteosa*, *dentine* and *enamel.* The first corresponds more closely with bone, being supplied with the *lacunæ* and small *haversien canals* when it occurs of sufficient thickness, and is found investing the fang of the tooth as it enters the alveolar process, and also forms the first covering of the temporary teeth. The *dentine* is of a firmer consistence from containing a greater proportion of inorganic matter, it corresponds with that structure known as ivory, in the tusk of the elephant. The *enamel* differs from both the other, by containing less animal matter, and is composed of solid prisms or fibres of extremely small dimensions, arranged side by side, and corresponding in length with the thickness of the layer which they form, the two surfaces being composed of the free extremities of the fibres, which are usually hexagonal.

As regards the composition of the tooth, it is first to be examined as containing two substances, *earthy* and *animal* matter. Chemical analysis of the front teeth or inscisors of the human subject give the following result:

| | Cementum. | Dentine. | Enamel. |
|---|---|---|---|
| Animal Matter | 29.27 | 28.70 | 3.59 |
| Earthy Matter | 70.73 | 71.30 | 96.41 |
| | 100 | 100 | 100 |

By a more complete analysis of the teeth may be obtained the earthy constituents that enter into their formation, and it will be observed that the different parts of the tooth differ chiefly as regards the amount of earthy particles which they contain.

The following analysis shows the components of the molar teeth of man, and of the bones of the arm and leg of a man of forty:

| | Dentine. | Enamel. | Bone. |
|---|---|---|---|
| Inorganic or earthy matter—Phosphate of lime with traces of fluate of lime | 66.72 | 89.82 | 54.61 |
| Carbonate of lime | 3.36 | 4.37 | 9.41 |
| Phosphate of magnesia | 1.08 | 1.34 | 1.07 |
| Salts, &c. | 83 | 88 | 2.35 |
| | 71.99 | 96.41 | 67.44 |
| Organic or animal matter | 28.01 | 3.59 | 32.56 |
| | 100 | 100 | 100 |

It will be seen from this analysis that there is a greater proportion of earthy matter entering into the formation of the teeth than bone, the enamel particularly, being composed almost entirely of earthy substances, there being only 3.59 parts of animal matter in the 100. The chief substance in the formation of both bone and tooth is the *phosphate of lime*, and it is found much more abundant in the latter, from which it may be inferred that to make the tooth strong and durable it must be supplied with a sufficient quantity of *lime*, particularly the *phosphate.* It is evident that should this substance be deficient in the nourishment of the fœtus, or the infant there must be an imperfect formation of the osseous system, and the teeth containing it in the greatest proportion will necessarily suffer first, and hence the caries and defects that are seen in these organs. If the deficiency be extreme the bones are imperfectly formed, and rachitis is the result. Thus it may be observed that the perfect organization of both depend upon a supply of the same material but in the former a small deficiency produces a more deleterious effect than in the latter.

The fœtus derives all its nourishment directly from the mother, and it is therefore necessary that her blood contain all the constituents required for the perfect development of the healthy child. This, by analysis, is found to be the case in healthy blood, and hence we see the importance of supplying the mother with good wholesome food, such as shall contain all the constituents required.

But there are instances, and I regret to say that they are not few, in which previous irregularities have perverted to a great extent the assimilating power of the mother, her constitution is enfeebled, and the sustenance which she can supply to the individual that will soon be thrust upon the world to suffer out a few brief years, and then fill an infant's grave, are barely sufficient to sustain the functions of life, and disease marks its earliest existence. Is there no remedy for this ? Are there no means by which even unhealthy mothers may produce healthy children ? I have no doubt but that this may, in a measure be accomplished, but the place to remedy this great evil is not with the present mother, but with the *children*, who are in a few years to be the fathers and mothers of a future generation.

If any reliance can be placed upon the analysis of the teeth, and of the substances required in their formation, it would be supposed that a healthy denture might be obtained in the infant by supplying to the mother's blood those substances which are deficient; the phosphate and carbonate of lime. Observation would seem to prove that this may be done. Dr. Taft relates a case in which the teeth of both parents were defective—their children were also affected with carious teeth almost from birth. During the mother's last gestation, she used considerable quantities of phosphate of lime, which was accompanied with the most favorable results. The teeth of the child were perfect, the eruptions prompt, and unaccompanied by any unfavorable symptoms. The temporary teeth remained sound until the time for the appearance of the permanent set, which were presented with great regularity.

This is a matter of vital importance to all who are, or expect to become mothers. Most of the mothers of the present day, in this country, are more or less diseased, and if it be true that a little medication will ensure an improvement in the organization of her offspring, that mother would be guilty of the grossest neglect, and deserve the censure of every friend of humanity, who would refuse to provide for the wants of her future child.

Much may be done to improve the condition of the unfortunate and unhealthy parents who are continually thrusting the innocent babes upon the cold charities of the world, as if their only and legitimate business was to obey the divine injunction to the fullest extent, regardless of the floods of disease and misery that must inevitably follow, as the result of their indiscretion. Anything that will improve the general health of the mother, will exert a like beneficial influence upon the constitution of the child, everything that adds to her comfort, and tends to relieve her from the cares and perplexities of life, will in a like manner result for the wellfare ofher offspring. The mother should not only be supplied with plenty of good whole some food, but during the whole period of gestation she should be surrounded by influences of the most pleasing kind, everything that would tend in the least to irritate her mind should be avoided, and associations of the most refined and elevater characted should be employed, such as will continually induce her to remember life as a blessing rather thanas a curse.

I know there are some who would look upon this as useless, or of small moment, but it is well known that even a mental emotion, a sudden burst of joy or anger, violent grief, or excessive fear on the part of the mother, may have such an effect as to materially modify the great functions of life, as absorption, assimilation, secretion, &c., and thus produce its deleterious effects upon the exotic that is dependent directly upon her for food.

Is it a matter of small moment that she has in her power the formation of a being that shall inhabit eternity, when any such excess or misdemeaner on her part, may stamp upon that character a peculiar conformation, that shall either cause it to fill a dishonored grave, or give it years of trial in after life to overcome the evil tendencies of an early and injurious impression? Not only has the mother control of the mind, but also of the body of her offspring, and her highest and most holy duty should be to provide, by every means in her power, for its wellfare.

So far as regards the use of medicinal agents, they should be of that character which are required in the growth of the various organs of the body, and as it has been shown the principle portion of the bones and teeth are composed of phosphate of lime or *bone earth*, as it is significantly designated, it is evident that the beneficial results would arise from its use in those instances where observation has proven it to be deficient.

Hence, I consider that the best and *only* means of procuring a healthy dentine, is in the power, and must be the work of the parent, let the mother repair, as far as possible, the broken constitution, supply herself with all the necessaries for the formation of perfect bone, blood and muscle, and if occasion requires, use daily any, or all of the earthy constituents that enter into the formation of those important organs, the teeth, and she may confidently expect that a healthy child will reward her efforts.

This is a matter that can be advanced only by close and extensive observation, and it is of the utmost importance that the members of the dental and medical professions shall investigate the subject as extensively as possible, so that instead of vague ideas, and uncertain hypotheses, we may have the lights of science to point out the certain and unerring means of relieving the human family of such a deleterious tendency.

But, to remedy the evil must be the work of the present and future generations, the *children* of to-day should be restrained from indulging in all the excesses that have ruined the parent, and allow only those substances which may result in the production of a healthy constitution; but this will be treated of more fully in a future paper, on the cultivation and preservation of good teeth.

